# Is It Necessary to Specifically Define the Cause of Surgically Treated Biliary Tract Infections? A Rare Case of* Raoultella planticola* Cholecystitis and Literature Review

**DOI:** 10.1155/2017/4181582

**Published:** 2017-05-07

**Authors:** Suat Can Ulukent, İnanc Samil Sarici, Nuri Alper Sahbaz, Yigit Mehmet Ozgun, Ozlem Akca, Kamuran Sanlı

**Affiliations:** ^1^Kanuni Sultan Suleyman Training and Research Hospital, Department of General Surgery, Istanbul, Turkey; ^2^Kanuni Sultan Suleyman Training and Research Hospital, Department of Clinical Microbiology, Istanbul, Turkey

## Abstract

*Raoultella planticola *is an aquatic and soil organism that does not notoriously cause invasive infections in humans. Infections in the literature are limited only in case reports. We present a very rare case of* R. planticola* cholecystitis. A 71-year-old female patient with abdominal pain was diagnosed with acute cholecystitis. Patient received intravenous antibiotic treatment, but the treatment failed and the patient underwent an open cholecystectomy. The final pathological result was gangrenous cholecystitis complicated with* R. planticola. *Eventually, the patient recovered with appropriate antimicrobial therapy. Patients with acute cholecystitis are usually treated without any microbiological sampling and antibiotic treatment is started empirically. To date, there have only been 5 reported biliary system related* R. planticola* infections in humans. We believe that* Raoultella *species might be a more frequent agent than usually thought, especially in resistant cholecystitis cases. Resistant strains should be considered as a possible causative organism when the patient’s condition worsened despite proper antimicrobial therapy. It should be considered safe to send microbiological samples for culture and specifically define the causative microorganisms even in the setting of a cholecystectomized patient.

## 1. Introduction

*Raoultella planticola* is an aquatic and soil organism that does not notoriously cause invasive infections in humans.* Raoultella planticola* infections are limited only in case reports. There have only been 5 reported cases of biliary tract related infections (cholecystitis, cholangitis, and pancreatitis) described so far in the English literature. Hereby, we report a case of cholecystitis complicated by* R. planticola.*

## 2. Case

A 71-year-old female patient is presented to the emergency department with the chief complaint of abdominal pain. She described a 1-week history of worsening right upper quadrant pain with nausea and vomiting. Her physical examination body temperature 37.5°C, pulse 90/min, blood pressure 120/80 mmHg, and respiratory rate 15/min. A slight distension and a tenderness in the right hypochondrium area were detected in the abdominal examination; abdominal defense and rebound phenomenon were absent. Her medical history revealed geophagia during childhood and a previous long-term dental treatment. She did not have diabetes or any prior surgeries.

White Blood Cell (WBC) count was 22.9  ×  10^9^/L [normal: 3.7  ×  10^9^/L–10  ×  10^9^/L], and C-reactive protein (CRP) was 150 (NV < 5). Ultrasound (USG) scan revealed a distended gall bladder with a thickened wall and multiple stones. The patient was diagnosed as acute cholecystitis and admitted to the surgical ward.

She received 3 days of intravenous (IV) cefazolin 2 × 1 gr/day. At the end of the 3rd day, her symptoms improved, but WBC and CRP levels remained high. On the 7th day of her treatment, her symptoms resolved and WBC levels normalized, but paradoxically CRP levels still remained high and control ultrasound revealed an increase in the thickness of the gall bladder wall and pericholecystic fluid collection. According to these findings, surgery was planned. During exploration, the gall bladder was found to be buried in the omentum with multiple foci of closed perforation. Cholecystectomy was performed and no complications occurred during the postoperative period. Cultures of microbiological sample were taken during the operation. The sample was cultivated on EMB and sheep blood agar with standard loop as the inoculant. Gram-negative bacilli grew on both culture media to a density of >100,000 CFU/mL. The isolate was analyzed by matrix-assisted laser desorption/ionization time-of-flight mass spectrometry using the VITEK MS (bioMérieux, Marcy l'Étoile, France) system and identified as* R. planticola.* Antimicrobial susceptibility testing was performed using the VITEK-2 compact system which was sensitive to cefazolin, ampicillin/sulbactam, ceftriaxone, ciprofloxacin, gentamicin, ceftazidime, tobramycin, and levofloxacin and resistant to ampicillin, cefuroxime, imipenem, trimethoprim/sulfamethoxazole, amoxicillin/clavulanic acid, cefuroxime axetil, and nitrofurantoin. According to the culture results, the ampicillin/sulbactam regimen was continued for 10 days and the patient was discharged with normal CRP levels and no complications. The final pathological result was gangrenous cholecystitis. In the 3rd postoperative month of follow-up, all the blood stats, USG, and magnetic resonance cholangiopancreatography (MRCP) images were normal.

## 3. Discussion

*Raoultella planticola *is a nonmotile, aerobic, Gram-negative bacillus. It was first described by Bagley et al. [1] as* Klebsiella planticola* in 1981 within environmental specimens and by Ferragut et al. [2] in 1983 as* Klebsiella trevisanii* with deoxyribonucleic acid (DNA) hybridization technique in soil and water specimens. Because of the DNA sequence similarities, in 1986, both microorganisms were merged within one species,* Klebsiella planticola,* by Gavini et al. [3].* R. planticola* carries a*β*-lactamase that makes this agent naturally resistant to several antimicrobial agents.

To date, there have only been 5 reported biliary system related infections in humans (Table 1). Alves et al. [4] in 2007 reported a case of acute pancreatitis and retroperitoneal abscess in a 45-year-old male. Yokota et al. [5] described a 65-year-old man developing septic shock and cholangitis caused by* R. planticola* in 2011. In 2012, Teo et al. [6] reported acute cholecystitis in a 62-year-old woman. In 2014, Salmaggi et al. [7] described a 70-year-old man with chronic obstructive pulmonary disease and pancreatic adenocarcinoma complicated with* R. planticola*-associated cholangitis and, in the same year, Ershadi et al. [8] reported a 49-year-old man presenting with acute cholecystitis with past medical history of alcohol abuse, alcoholic cirrhosis, and diabetes mellitus. Most of the infected cases in literature either have significant comorbidities or have a history of trauma or an invasive procedure, but there is no trauma history or comorbidity seen in our case.

*Antibiotic treatment is started empirically with agents covering enterococci and *Enterobacteriaceae*, including Escherichia coli.* Generally, acute cholecystitis cases are expected to be clinically regressed after parenteral antibiotic treatment. If the clinical regression does not occur within 2 days, it is considered resistant cholecystitis. We believe that* Raoultella* species might be a more frequent agent then usually thought, especially in resistant cholecystitis cases.* R. planticola* has the ability to become antibiotic-resistant trough plasmid genes. Most of the cases reported in the literature are severe cases of infection or sepsis. Therefore, resistant strains should be considered as a possible causative organism when the patient's condition worsens despite proper antimicrobial therapy.

## 4. Conclusion

Potential risk factors for* R. planticola* include invasive medical procedures, immunocompromised patients, significant comorbidities, and trauma with soil contamination.

We believe that resistant strains may also cause serious infections in the absence of these predisposing factors. Despite the fact that our patient did not have any risk factors and had severe case of cholecystitis, it is still prudent to consider* R. planticola* as a possible causative agent among patients with these risk factors. Given its resemblance to* Klebsiella* species, this bacterium has the potential to become multidrug resistant and cause increased numbers of mortality and morbidity. In order to prevent this, it should be viewed as an invasive organism, requiring proper treatment, rather than a noninfectious member of the flora. Hence, it should be considered safe practice to send microbiological samples for culture and specifically define the causative microorganisms even in the setting of a cholecystectomized patient.

## Figures and Tables

**Table 1:** Biliary tract related infections caused by *R. planticola, *reported in the literature.

Author	Patient	Clinical manifestation	Comorbidity	Trauma/invasive procedure	Immune deficiency	Treatment
Alves et al.	45/male	Pancreatitis and retroperitoneal abscess	Alcoholism, pneumonia	No	Yes	Imipenem, amikacin

Yokota et al.	65/male	Cholangitis	Adenocarcinoma of the neck	ERCP^*∗*^	Yes	Piperacillin, tazobactam

Teo et al.	62/female	Acute cholecystitis	Celiac disease	No	No	Amoxicillin-clavulanate

Salmaggi et al.	70/male	Acute cholecystitis	Pancreatic adenocarcinoma	ERCP, biliary stenting	Yes	Ciprofloxacin, metronidazole

Ershadi et al.	49/male	Acute cholecystitis	Alcoholism, alcoholic cirrhosis, diabetes mellitus	Percutaneous cholecystostomy	Yes	Tigecycline

^*∗*^ERCP: endoscopic retrograde cholangiopancreatography.
